# Prokaryotic Aquaporins

**DOI:** 10.3390/cells8111316

**Published:** 2019-10-24

**Authors:** Huichun Tong, Qingqing Hu, Lin Zhu, Xiuzhu Dong

**Affiliations:** 1 State Key Laboratory of Microbial Resources, Institute of Microbiology, Chinese Academy of Sciences, No.1 Beichen West Road, Chaoyang District, Beijing 100101, China; 2 University of Chinese Academy of Sciences, No.19A Yuquan Road, Shijingshan District, Beijing 100049, China

**Keywords:** aquaporins, facilitated diffusion, prokaryote, selective filter, tetramer, hydrogen peroxide

## Abstract

Aquaporins are integral membrane proteins that facilitate the diffusion of water and other small, uncharged solutes across the cellular membrane and are widely distributed in organisms from humans to bacteria. However, the characteristics of prokaryotic aquaporins remain largely unknown. We investigated the distribution and sequence characterization of aquaporins in prokaryotic organisms and summarized the transport characteristics, physiological functions, and regulatory mechanisms of prokaryotic aquaporins. Aquaporin homologues were identified in 3315 prokaryotic genomes retrieved from the Kyoto Encyclopedia of Genes and Genomes (KEGG) database, but the protein clustering pattern is not completely congruent with the phylogeny of the species that carry them. Moreover, prokaryotic aquaporins display diversified aromatic/arginine constriction region (ar/R) amino acid compositions, implying multiple functions. The typical water and glycerol transport characterization, physiological functions, and regulations have been extensively studied in *Escherichia coli* AqpZ and GlpF. A *Streptococcus* aquaporin has recently been verified to facilitate the efflux of endogenous H_2_O_2_, which not only contributes to detoxification but also to species competitiveness, improving our understanding of prokaryotic aquaporins. Furthermore, recent studies revealed novel regulatory mechanisms of prokaryotic aquaporins at post-translational level. Thus, we propose that intensive investigation on prokaryotic aquaporins would extend the functional categories and working mechanisms of these ubiquitous, intrinsic membrane proteins.

## 1. Introduction

Aquaporins are integral membrane proteins that form tetramers and facilitate the diffusion of water and some small, uncharged solutes across cellular membranes [1,2]. First found in human red cell membranes [1], aquaporins have subsequently been shown to be widely distributed in all living organisms [3,4,5]. Phylogenetically, aquaporins belong to the major intrinsic protein (MIP) family that is comprised of more than 1700 integral membrane proteins [6,7]. Based on the primary sequences, they are classified into the water-selective aquaporins (AQPs), glycerol-transporting aquaglyceroporins (GLPs) and unorthodox aquaporins; the third subfamily is only present in animals with unverified substrate permeability [3,8]. All aquaporins possess two characteristic Asn–Pro–Ala (NPA) motifs, which meet at the middle of the channel and form a constricted region serving as a size selective filter, but different conservative amino acid residues constitute the aromatic/arginine constriction region (ar/R region), which is also known as the selective filter (SF) that facilitates substrate transport [3,9,10]. The ar/R region of AQPs is composed of F(H/I)XR, whereas that of GLPs is WG(F/Y)R [9]. Aquaporin-1, an AQP in human red blood cells, is the first reported water facilitator that speeds up transmembrane influx and efflux of water [1]. Since then, more AQP and GLP homologues have been found and extensively studied in mammals and plants [11,12,13,14,15,16]. To date, 13 and as high as 120 aquaporin isoforms have been identified in mammals and plants, respectively [10,17,18]. The plant AQPs especially, display extensive diversifications; phylogenetically they are divided into five major sub-families: plasma membrane intrinsic proteins (PIPs), tonoplast intrinsic proteins (TIPs), NOD26-like intrinsic proteins (NIPs), small basic intrinsic proteins (SIPs), and uncharacterized intrinsic proteins (XIPs) [3,19]. Aquaporins exhibit high selectivity and efficiency on water or glycerol permeation [11,16,17,18]. In addition, a number of other uncharged solutes or gases are reported to cross the cellular membrane via aquaporin channels, which include urea, ammonia, hydrogen peroxide (H_2_O_2_), carbon dioxide (CO_2_), metalloids, nitric oxide (NO), malate-aluminum, and even ions [20,21,22,23,24,25,26]. Because these small molecules are involved in a variety of metabolic processes or function as signal molecules [13,27], aquaporins have been verified to play important roles in organisms in stress response, growth, development, and disease processes, including tumorigenesis and metabolic disorders [28,29,30,31]. Consequently, aquaporins are currently considered potential drug targets for disease prevention and treatment in humans [32].

Although genome sequencing indicates that most of the prokaryotic species carry water or glycerol-transporting aquaporin homologues, their functions remain largely unknown [4,33,34]. A recent study revealed that an aquaporin from catalase-negative *Streptococcus* facilitates the efflux of endogenous H_2_O_2_, and thus, plays important roles in H_2_O_2_ detoxification and also inter or intra-species competition [35]. By using genome sequence information and homology analysis, we investigated the distribution and sequence characteristics of aquaporin homologues in prokaryotic species, and summarized the findings of recent investigations on prokaryotic aquaporins in relation to substrate transport, physiological functions, regulatory mechanisms, and the factors contributing to the stability of aquaporin tetramers.

## 2. Distribution and Phylogeny of the Aquaporin Homologues in Prokaryotes

Prokaryotic aquaporins were first recognized in *Escherichia coli*, in which two orthologs, namely, *aqpZ* and *glpF*, are phylogenetically related to mammalian and plant water and glycerol channels, respectively [36,37]. Using *aqpZ* and *glpF* as probes to query the sequenced genomes of 5294 bacteria and 299 archaea in the Kyoto Encyclopedia of Genes and Genomes (KEGG) database (latest updated on September 11, 2019), orthologs of water-type and glycerol-type aquaporins were found in 3315 prokaryotic species. Of these, 977 bacterial species encode both AQPs and GLPs, whereas 698 bacteria only encode AQPs, such as *Veillonella rodentium* and *Acidaminococcus fermentans*, and 1552 only encode GLPs, such as *Pectobacterium atrosepticum*, *Dichelobacter nodosus*, *Aerococcus urinae*, and *Streptomyces coelicolor*. However, 2067 bacterial species do not harbor any aquaporin homologues, including all of the species affiliated with phyla Fibrobacteres and Lentisphaerae. Some animal pathogenic microorganisms and extreme-environment inhabitants do not carry a single known AQP ortholog in their genomes. This could be because they either encode currently undefined aquaporins or employ other apparatus for water and solute transport. In contrast, aquaporin orthologs are only distributed in a few archaeal phyla. Nineteen water-type AQPs appear to be restricted to Euryarchaeota and Thaumarchaeota, and 69 GLP orthologs were only found in Euryarchaeota and Crenarchaeota. None of the aquaporin orthologs were found in the genomes of the phyla of Nanoarchaeota, Micrarchaeota, Korarchaeota, Bathyarchaeota, and Lokiarchaeota, and this may be attributable to the non-complete genome data that were assembled from the metagenomes. Of note, human AQP11 and AQP12, which belong to a third subfamily of aquaporins and are only present in animals, were used as query sequences to search the prokaryotic genome database, but no homologues could be identified, indicating that prokaryotic organisms do not encode unorthodox aquaporin homologues.

**Figure 1:**
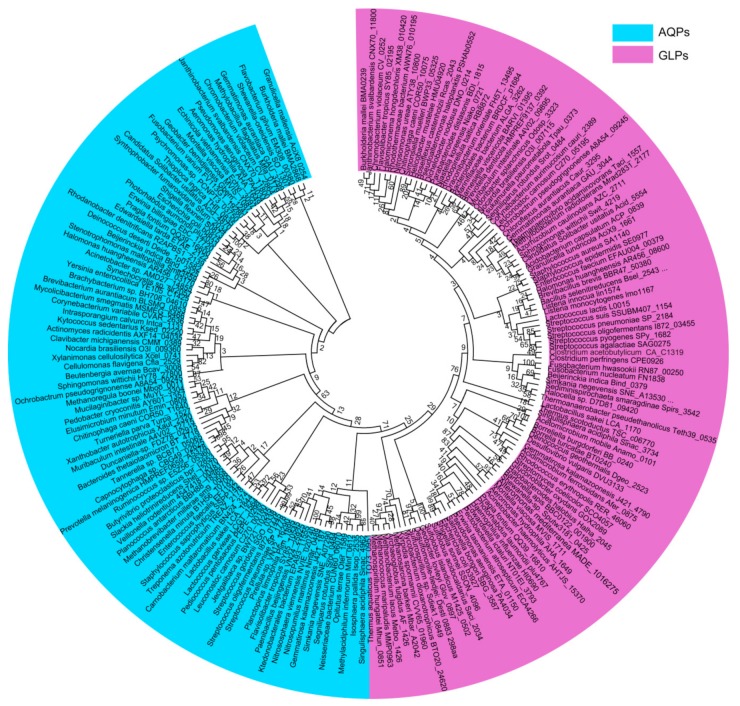
Phylogenetic analysis of the prokaryotic aquaporins. The 94 water-transporting aquaporins (AQPs) (blue color) and 103 glycerol-transporting glycerol-transporting aquaglyceroporins (GLPs) (purple color) are from representative prokaryotic organisms. Amino acid sequences were retrieved from UniProt. CLUSTALW implemented in MEGA7 was used for multiple sequence alignment and the phylogenetic tree was constructed using the maximum likelihood (ML) method with bootstrap values of 1000 replicates.

**Figure 2:**
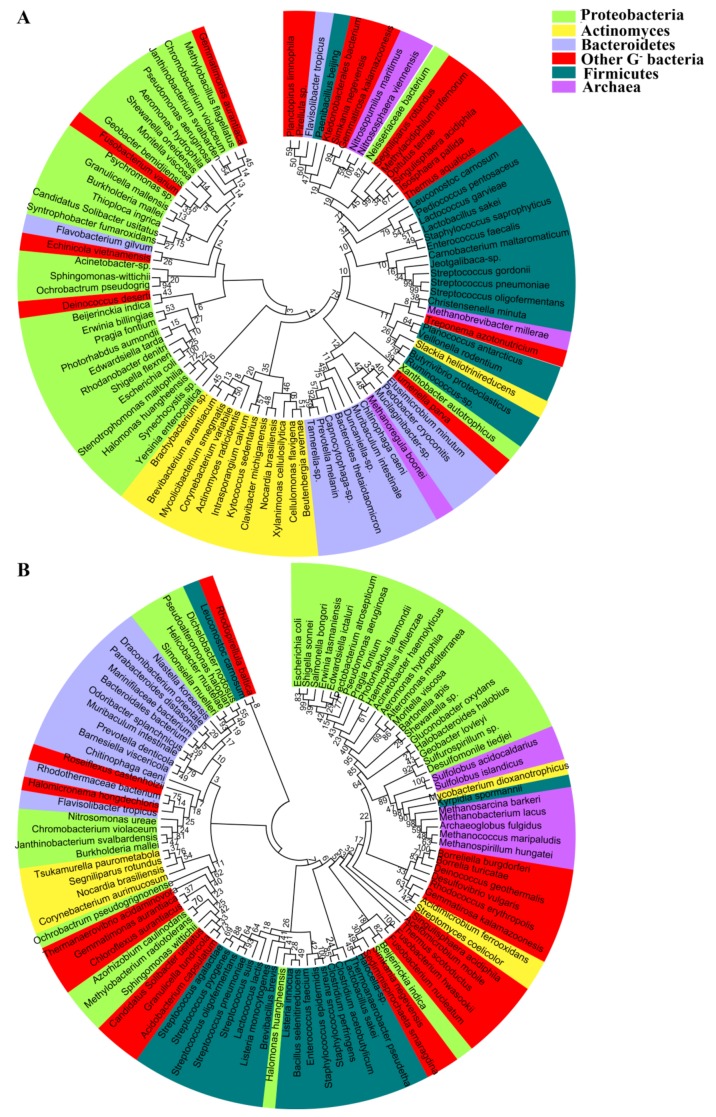
Phylogenetic analysis of 94 water-transporting AQPs (**A**) and 103 GLPs (**B**) from representative prokaryotic organisms. The amino acid sequences were retrieved from UniProt. CLUSTALW implemented in MEGA7 was used for multiple sequence alignment and the phylogenetic tree was constructed using the maximum likelihood (ML) method with bootstrap values of 1000 replicates.

Phylogenetic analysis of the prokaryotic aquaporins, including 94 AQPs and 103 GLPs from the representative prokaryotic species, shows that they are clustered into two major clades; namely, the water-transporting AQPs and the glycerol-facilitating GLPs (Figure 1). This division is concordant with different substrates they permeate. In general, the AQP clustering pattern coincides with the phylogeny of the species carrying them. However, some within-individual clades are discordant, such as the Actinobacteria AQPs, which are even more closely related to Proteobacteria than Firmicutes AQPs (Figure 2A). The phylogeny of the prokaryotic glycerol facilitator GLPs is even more conflicting with that of species harboring them, in which the clustering of GLPs from various phyla of Gram-negative bacteria is mixed, and those from Gram-positive and Gram-negative bacteria are not distinctly separated (Figure 2B). In addition, the archaeal AQPs and GLPs are clustered with their counterparts from Gram-negative bacteria. Therefore, this suggests that aquaporins, which exhibit the most conserved and fundamental physiological functions, could have been originated at a time predating the divergent evolution of the prokaryotic species and could have been lost or gained through extensive horizontal gene transfers during species evolution. Horizontal aquaporin gene transfer could have also occurred in eukaryotes, such as for the AQP paralogues, which can be highly dissimilar in a single organism, although all of the eukaryotic AQPs are supposed to have evolved from prokaryotic AQP channels [38].

## 3. The Conserved Amino Acid Motifs and Topological Characteristics of Prokaryotic Aquaporins

**Figure 3:**
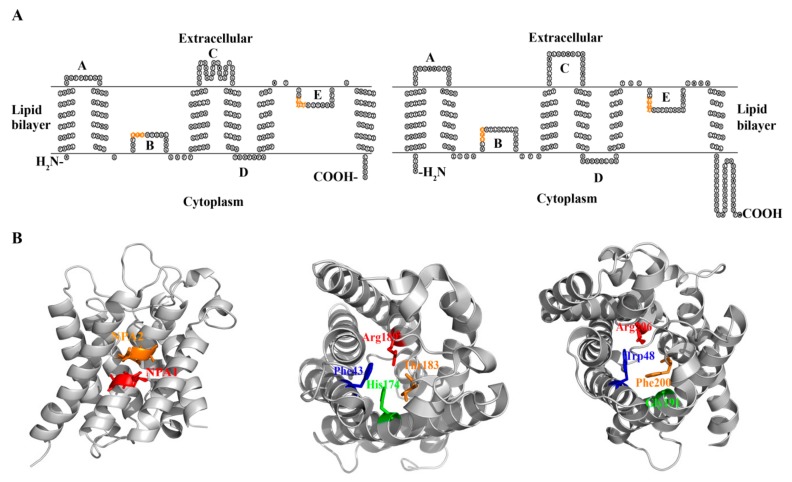
The membrane topology and conserved motifs of aquaporins from representative prokaryotic organisms. (**A**) *Escherichia coli* AqpZ (left) and human aquaporin-1 (right) display the same membrane topology, but AqpZ has a significantly lower number of intracellular N and C-terminal amino acid residues. *E. coli* GlpF possesses the same topological structure as AqpZ, and thus, is not shown. Orange letters represent NPA amino acid residues on loops B and E. (**B**) Structural modeling shows the two NPA motifs meet at the central region of the AqpZ/GlpF channels (left); the conserved aromatic/arginine constriction region (ar/R) selective filter residues Phe43, His174, Thr183, Arg189 of AqpZ (middle) and Trp48, Gly191, Phe200, and Arg206 of GlpF (right) are shown by sticks.

The *E. coli* water channel *aqpZ* and glycerol facilitator *glpF* are the most intensively studied prokaryotic aquaporins, and they encode 231 and 281-amino acid polypeptides, respectively [36,37]. Resembling their eukaryotic counterparts, both AqpZ and GlpF are proteins comprised of six transmembrane domains (H1–H6), three extracellular loops (A, C, and E), and two cytoplasmic loops (B and D). Loops B and E are highly hydrophobic and insert into the lipid bilayer from opposite directions (Figure 3A). The two NPA motifs, located at loops B and E respectively, meet in the middle of the lipid bilayer and form a substrate-permeable channel (Figure 3B) [39,40].

**Figure 4:**
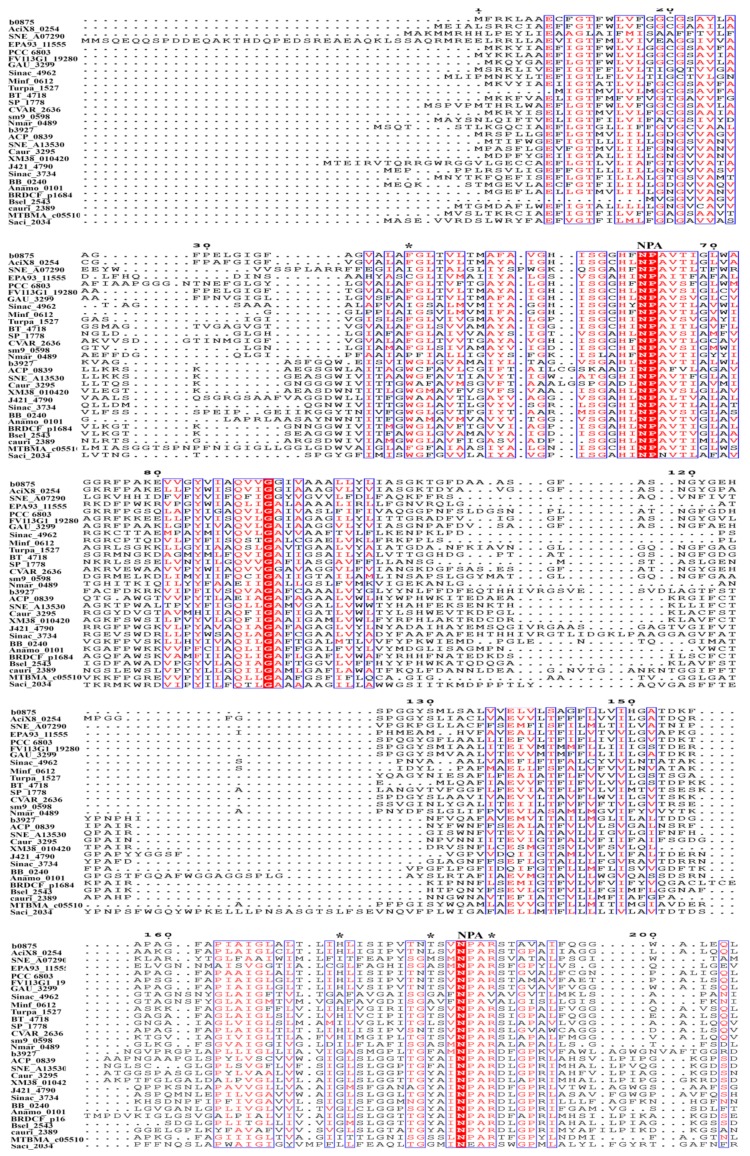
Amino acid sequence alignment of the AQPs and GLPs from representative prokaryotic species. The protein sequences were retrieved from UniProt, and alignment was conducted using Clustal Omega. The ar/R selective filter residues in AQPs and GLPs are labeled with asterisks, and the two NPA motifs are labeled on the top. b0875, *Escherichia coli*; AciX8_0254, *Granulicella mallensis*; SNE_A07290, *Simkania negevensis*; EPA93_11555, *Ktedonobacterales bacterium*; PCC_6803, *Synechocystis sp*.; FV113G1_19280, *Fusobacterium varium*; GAU_3299, *Gemmatimonas aurantiaca*; Sinac_4962, *Singulisphaera acidiphila*; Minf_0612, *Methylacidiphilum infernorum*; Turpa_1527, *Turneriella parva*; BT_4718, *Bacteroides thetaiotaomicron*; SP_1778, *Streptococcus pneumoniae*; CVAR_2636, *Corynebacterium variabile*; sm9_0598, *Methanobrevibacter millerae*; Nmar_0489, *Nitrosopumilus maritimus*; b3927, *Escherichia coli*; ACP_0839, *Acidobacterium capsulatum*; SNE_A13530, *Simkania negevensis*; Caur_3295, *Chloroflexus aurantiacus*; XM38_010420, *Halomicronema hongdechloris*; J421_4790, *Gemmatirosa kalamazoonesis*; Sinac_3734, *Singulisphaera acidiphila*; BB_0240, *Borreliella burgdorferi*; Anamo_0101, *Acetomicrobium mobile*; BRDCF_p1684, *Bacteroidales bacterium*; Bsel_2543, *Bacillus selenitireducens*; cauri_2389, *Corynebacterium aurimucosum*; MTBMA_c05510, *Methanothermobacter marburgensis*; Saci_2034, *Sulfolobus acidocaldarius*.

Sequence alignment of AqpZ, GlpF, and their homologues, including 94 AQPs and 103 GLPs, which are used for phylogenetic analysis, was performed using the Clustal Omega, and the sequence alignment of 15 AQPs and 14 GLPs, which are the most representative of the overall characterization of prokaryotic aquaporins, are shown in Figure 4. The two characteristic NPA motifs are highly conserved among bacterial aquaporins. Non-conserved NPAs are found in a *Segniliparus rotundus* AQP, in which the N-terminal NPA motif is replaced by TPV, and the *Flavisolibacter tropicus* and *Segniliparus rotundus* AQPs, in which the C-terminal NPA motifs are substituted by NPI and VPA, respectively. Residue-substituted NPAs were also observed in some bacterial GLPs, such as the N-terminal NPS in *Bartonella apis*, NPI in *Burkholderia mallei*, and NPV in *Janthinobacterium svalbardensis*; and the C-terminal NAA in *Bartonella apis*; SPA in *Burkholderia mallei*, *Janthinobacterium svalbardensis*, and *Chromobacterium violaceum*; NLA in *Leuconostoc carnosum*; and NPV in *Corynebacterium aurimucosum*.

Consistent with the divergent phylogenetic clustering and different facilitated substrates, AqpZ and GlpF possess four distinct conserved amino acid residues that comprise the ar/R region, which is also named the selective filter (SF) (Figure 3B) [39,40,41,42]. The ar/R region of the AqpZ homologues in bacteria is mostly composed of a Phe at the second transmembrane helix (H2); a His or Ile at the fifth transmembrane helix (H5); a Cys, Thr, Ala, Leu, Gly, or Val at loop E (LE1), which provides a backbone carbonyl oxygen; and an Arg residue in most species, but that is substituted by Val in six species, at loop E (LE2), which provides donor hydrogen bonds for water molecules. Based on the hydrophilic characteristics of the amino acid residues in the SF region, these AQPs are predicted as water facilitators. However, IAGV constitutes the SF region of an AQP in *Singulisphaera acidiphila* belonging to phylum Planctomycetes, and *Isosphaera pallid*, *Methylacidiphilun infernorum*, and *Opitutus terrae* of phylum Verrucomicrobia; AIGV is in the *Neisseriaceae bacterium* AQP. Because only glycine is hydrophilic in this IAGV/AIGV region, whether these AQPs function as water facilitators remains unclear. The SF of GlpF and its bacterial homologues is mostly composed of a Trp at H2; a Gly in most species, but that is substituted by Ile in five species, at H5; a Phe, Tyr, Ala, or Ser at position LE1; and an Arg residue at LE2. Overall, the SF amino acid residues of the GLPs are more hydrophobic than those of the AQPs, in accordance with the characteristics of the facilitated substrates.

Similar to eukaryotic aquaporins, both the N and C-terminal amino acid residues of AqpZ and GlpF are localized in the cytoplasm [36,39,40]. However, the lengths of the AqpZ cytoplasmic domains are significantly shorter than its eukaryotic counterpart aquaporin-1 (Figure 3A) [39,43], implying that the AqpZ protein is more hydrophobic. This could explain why the *E. coli* AqpZ tetramers, unlike the eukaryotic aquaporins, resist dissociation on an SDS-PAGE gel [44].

The SF region of the archaeal AQPs is similar to its bacterial counterparts, but for GLPs, except for an Arg at LE2, the other three conserved amino acids are different from those of bacteria (Figure 4). For example, a Phe at H2, a medium-sized and hydrophobic Ile or Val at H5, and a Ser or Ala at LE1 are found in Euryarchaeota GLPs. However, in the GLPs of *Sulfolobus* spp. of Crenarchaeota, a Trp at H2, a Lys at H5, and a Gly at LE1 are observed, and the N and C-terminal NPA motifs are replaced by NPN and NEA, respectively. Thus, it appears that the archaeal GLPs merge the characteristics of narrow SF and channel in the obligate water-type aquaporins with the more hydrophobic but less polar SF of the aquaglyceroporins. A consensus SF sequence to that of the archaeal GLPs occurs in some bacterial GLPs, such as those from *Geobacter lovleyi*, *Desulfomonile tiedjei*, and *Kyrpidia spormanii*. AqpM, an archaeal GLP in *Methanothermobacter marburgensis*, has been experimentally verified to weakly facilitate water permeation, but not glycerol [45]. Thus, archaeal GLPs could represent primitive non-specialized aquaporins, a possible ancestor of specialized aquaporins [45].

## 4. The Protein Structures and Substrate Selectivities of Prokaryotic Aquaporins

The higher-resolution atomic structures of AqpZ and GlpF facilitate elucidating the mechanisms of transport and substrate selectivities in prokaryotic aquaporins [39,40]. Protein structures reveal that both AqpZ and GlpF form tetramers, which are comprised of four monomers, each containing an ar/R region and two NPA motifs located at the ends of two half helices (Figure 3B). The ar/R region is situated near the extracellular exit and forms the narrowest point of the substrate channel. The AqpZ ar/R region consists of three hydrophilic amino acids, His174, Thr183, and Arg189, and a hydrophobic Phe43 (Figure 3B), and they form a pore with a diameter of ~4 Ǻ, which is close to the diameter of H_2_O molecule. Therefore, the ar/R region-formed pore size and characteristics of amino acid in the channel determine AqpZ to specifically select water, which is supported by molecular dynamic simulation [46].

The protein structure and energy calculation also provide insights into the molecular mechanisms of GlpF for its specificity on glycerol. The 2.2-Angstrom resolution crystallized structure of the glycerol-bound *E. coli* GlpF indicates that the restrictive ar/R region consists of side chains of Trp48, Gly191, Arg206, and the carbonyl of Phe200, and the channel radius formed is ~2.5 Ǻ in width. The glycerol alkyl backbone wedges into this hydrophobic corner, while the successive hydroxyl groups form hydrogen bonds with a pair of acceptor and donor atoms. This structure elucidates the mechanism of selective permeability for linear carbohydrates but excludes ions [40]. Further, a glycerol-mediated “induced fit” gating motion was proposed for GlpF to select glycerol over water [47]. A subsequent molecular dynamics simulation has found that, in addition to glycerol, GlpF is also permeable to both water and small solutes, such as urea [48,49]. Therefore, it is the water channel, not the solute channel, which determines the substrate selectivity of an aquaporin; i.e., an aquaglycerolporin may facilitate both glycerol and water’s transport, whereas an aquaporin exclusively facilitates water’s transport [49].

The only experimentally studied archaeal aquaporin AqpM, which is from *Methanothermobacter marburgensis*, also forms tetramers, and its SF or ar/R region is composed of F62, I187, S196, and R202. AqpM has a channel radius of ~1.4 Å, resembling that of a water molecule [45,50,51]. Unlike *E. coli* AqpZ, the AqpM SF contains a hydrophobic Ile instead of a hydrophilic His, which is a key residue in AqpZ for water selectivity. Therefore, AqpM possesses a hybrid characteristic of the narrow channel radius of AqpZ and the more hydrophobic SF of GlpF. Accordingly, a molecular dynamics simulation suggested that AqpM has lower water permeability than *E. coli* AqpZ but greater than GlpF, whereas it has a significantly lesser glycerol permeability than GlpF [52]. Therefore, the hybrid features of the archaeal aquaporin AqpM between the two well-known aquaporin families indicate that it could represent a novel family of aquaporins.

## 5. Research Approaches Used for Determining the Transport Properties of Prokaryotic Aquaporins

Although protein structure and molecular dynamics simulations have provided conformational and dynamic data of aquaporins in transporting substrates, experimental evidence is essential to confirm the transporting activities [53]. For that, both in vitro biochemical and ex vivo heterologous expression approaches have been developed. Specifically, the membrane protein aquaporins are reconstituted within liposomes to generate proteoliposomes [54]; alternatively, the cDNAs are heterologously expressed in *Xenopus laevis* oocytes, which are almost not permeable for water, glycerol, or other solutes [1]. In addition, yeast strains, which have different membrane lipid components from those of *X. laevis* oocytes, are also employed as surrogate hosts to study aquaporin transporting characteristics [55].

By means of the above approaches, the capability of AqpZ in facilitating water diffusion is evaluated by swelling of *X. laevis* oocytes or proteoliposomes that result from external hypo-osmotic stress [1,56]. Determining the glycerol permeability of GlpF is done by applying an inward glycerol gradient to cells/proteasomes, and glycerol transport function could be evaluated by observing the swelling of cells/proteasomes [53,54,57]. This is based on the principle that an extracellular higher concentration of glycerol will cause an osmotic, stress-driven water efflux, and thus, temporary cell shrinking, but accompanying solute and water influx, cells/proteoliposomes swell again. Therefore, GLPs facilitating glycerol influx will result in a cell shape transition from shrinking to swelling. By subtracting the volume change rate of the vacant cells/proteoliposomes, the osmotic permeability (Pf) and solute permeability (Ps) coefficients of aquaporins can be obtained. Another parameter that presents the activity facilitated by an aquaporin is the lower activation energy (Ea) for water or solute fluxing through a hydrophilic channel than across a hydrophobic lipid bilayer [54]. Ea can be obtained by evaluating Pf or Ps along temperature changes [44]. Water or solute influx causing cell volume change can be monitored by optical detection systems, such as light transmission [58], absorbance [59], scattering [60,61,62], or stopped-flow spectroscopy [63]. Volume-sensitive fluorescent dyes or genetically encoded fluorescent proteins are frequently employed to monitor cellular volume changes as well [64,65,66,67]. In addition, isotope-labeled solutes are used as markers that directly indicate aquaporin-facilitated solute influx across cellular membranes, which can be monitored by a liquid scintillation counter [68].

Upon heterologous expression of the gene in *Xenopus* oocytes or reconstituting the protein in proteoliposomes, the *E. coli* AqpZ shows very high activity in facilitating water permeability and requires low activation energy [44]. However, the experiments did not show that AqpZ transports nonionic solutes, such as urea and glycerol, demonstrating that it is an exclusive water facilitator [39,44]. Similarly, the *E. coli* GlpF in glycerol transport has been determined by injecting the mRNA into *X. laevis* oocytes [69,70]. In comparison to the vacant oocytes, GlpF increased cellular uptake to up to >200 mM glycerol and displayed a lower activation energy (Ea = 4.5 kcal/mol) [70], thereby supporting the prediction of Heller et al. (1980) [68] that GlpF mediates glycerol diffusion via a pore-type mechanism. Similar to eukaryote homologues, mercuric ion (Hg^2+^) blocks GlpF in the transport of glycerol by inactivating the cysteine residue situated in the substrate channel [70]. This indicates that prokaryotic GLPs employ the same transport mechanism as eukaryotic orthologs.

More than 30 years of studies on eukaryotic aquaporins reveal that the substances facilitated by aquaporins are not limited to water and glycerol, but include other small molecules, such as H_2_O_2_, and even gas molecules, including CO_2_, O_2_, and NO [21,23,25]. Similarly, cells and proteoliposomes can also be used in study of aquaporins in facilitating gas permeation; for example, CO_2_ transport can be monitored by determining cellular carbonic acid accumulation or measuring stable-isotope labeled CO_2_ [23]. O_2_ transmembrane permeation can be monitored using a dissolved oxygen electrode [25] or phosphorescent oxygen probe [71]; its fluorescence will be quenched as oxygen concentration decreases; alternatively, it can be done using hemoglobin as an intracellular oxygen indicator [23]. It is well-known that high concentrations of H_2_O_2_ may be harmful to cells, but lower concentrations of H_2_O_2_ function as signaling molecules that modulate important cellular physiological behaviors [13,27]. Fluorescent dyes and the genetically encoded fluorescent protein HyPer have been developed to specifically detect intracellular H_2_O_2_ [27,72,73]. Compared with the fluorescent dyes, HyPer estimates the cellular H_2_O_2_ levels in a reversible and real-time manner [74,75]. HyPer is constructed by inserting the yellow fluorescent protein cpYFP into the regulatory domain of the *E. coli* H_2_O_2_-sensing protein OxyR. H_2_O_2_ oxidizes Cys199 and Cys208 on the OxyR regulatory domain to form a disulfide bond, and the structural change in HyPer protein causes HyPer to emit green fluorescence [72]. Using HyPer as an intracellular H_2_O_2_ reporter, several aquaporins in mammals and plants have been verified to transport H_2_O_2_ across the cellular membrane [13,27,76]. Although the molecular mechanisms of eukaryotic aquaporins in transporting H_2_O_2_ remain unclear, H_2_O_2_ is considered to be transported through the H_2_O channel due to the two having similar sizes and electrochemical properties [77].

*Streptococcus oligofermentans* is a catalase-negative, facultatively anaerobic Gram-positive bacterium and is well-known for producing and tolerating high concentrations of H_2_O_2_ [78,79]. In a previous study, we found that H_2_O_2_ induced the expression of *So*-*aqpA*, a water-transporting aquaporin gene of *S. oligofermentans*, implying that So-AqpA could be involved in H_2_O_2_ permeation. Using HyPer as a cellular H_2_O_2_ reporter, we found that So-AqpA functions as a peroxiporin to facilitate bidirectional H_2_O_2_ permeation across *S. oligofermentans* cell membrane. Moreover, the heterologous expression of So-aqpA accelerates exogenous H_2_O_2_ influx into *Saccharomyces cerevisiae* and *E. coli* cells [35]. Therefore, a novel function was found for a bacterial aquaporin.

## 6. Physiological Functions of Prokaryotic Aquaporins

Because influxes or effluxes of water and solutes through cell membranes are an essential action in osmoregulation of organisms, the physiological functions of the prokaryotic aquaporins have been extensively studied in *E. coli*. Cryoelectronic microscopy observed that *E. coli* AqpZ mediated a rapid influx or efflux of water when the bacterium encountered a sudden down and upshift of extracellular osmolarity [80], indicating the important roles of AqpZ in osmoregulation. AqpZ also contributes to volume expansion in the rapidly growing mid-exponential cells. Consequently, the absence of AqpZ significantly reduced the competitive viability of the *E. coli* wild strain at lower osmolarity [81].

Similarly, deletion of the *glpF* gene leads to reduced growth of *E. coli* in lower concentrations of glycerol [82], indicating that the facilitator is important for the efficient uptake of glycerol, particularly at lower contents. Uptake and phosphorylation of glycerol are closely coupled actions, consistent with the clustered gene organization of *glpF* and *glpK*, with the latter encoding glycerol kinase. However, with high concentrations of glycerol, passive diffusion through cellular membrane contributes the major portion of the influx, as the *glpF* mutant maintains good growth [82]. However, GlpF appears to be essential to *Pseudomonas aeruginosa*, as knocking out the *glpFK* genes inhibits the bacterial growth when using glycerol as the sole carbon source [83]. This implies that cellular membrane characteristics influence glycerol permeation.

Lactic acid bacterium *Lactobacillus plantarum* aquaporins are also verified to export the metabolic end products along a concentration gradient [84]. *L. plantarum* is a facultatively anaerobic bacterium and gains ATP by fermenting glucose to produce lactic acid. It encodes six glycerol-transporting aquaporins, GlpF1–GlpF6. Heterologous expression in *X. laevis* oocytes has revealed that GlpF2, GlpF3, and GlpF4 facilitate transmembrane diffusion of water, dihydroxyacetone, and glycerol, respectively. In addition, GlpF1 and GlpF4 facilitate efflux of D/L-lactic acid, and the double gene mutant shows retarded growth under mild lactic acid stress. The lactic acid transport capacity of GlpF1 and F4 is conserved in the order of Lactobacillales; therefore, new substrates have been found in prokaryotic aquaporins.

*S. oligofermentans* So-AqpA, a water-type aquaporin, functions as a peroxiporin that facilitates the efflux of endogenous H_2_O_2_; thus, playing a detoxification role that protects the bacterium from oxidant attack [32]. In addition, So-AqpA-mediated H_2_O_2_ efflux endows its intra and interspecies competitions over the dental caries pathogen *Streptococcus mutans*. Of note, the *So*-*aqpA* orthologs and the functionally important Phe40 are present in all of the streptococcal species, implying that this H_2_O_2_-detoxifying mechanism could be widely used by streptococci [35].

## 7. Mechanisms of the Regulatory Expression of Prokaryotic Aquaporins

Regulated expression of aquaporins, which could occur at either the transcriptional or post-translational level, is critical to the osmoregulation and solute homeostasis in microorganisms and mammals [85,86]. Eukaryotic water selective AQPs are frequently regulated by a post-translational gating mechanism, which controls the aquaporin flux rate by maintaining pore conformations at open or closed status or by trafficking, whereby the AQPs are shuttled from the intracellular storage sites to the cytoplasmic membrane [87]. Regulatory factors, such as phosphorylation [14], pH [88], divalent cations [89], and membrane surface tension [90], have been shown to regulate the gating behavior of yeast, plant, and mammalian AQPs.

To date, the regulatory mechanism of bacterial aquaporin expression has been observed at the transcriptional level, and in general, expression of an aquaporin gene is frequently induced by its substrate. The *E. coli aqpZ* gene is induced by a hypo-osmotic circumstance [81], and H_2_O_2_ induces the transcription and translation of the *S. oligofermentans So*-*aqpA*, which encodes a dedicated peroxiporin. Two redox-regulatory transcriptional repressors, PerR (peroxide responsive repressor) and MntR (manganese transporting repressor) regulate the *So*-*aqpA* expression in response H_2_O_2_ [35].

The gating mechanism has been considered to regulate the substrate transport of AqpZ [91]. Initially, the crystallized structure found that R189, a conserved residue in SF region of AqpZ, could serve as a gate that shifts the monomeric water pore conformation to an open or closed status, and a molecular dynamics simulation also observed a rapid switch from open to closed conformations of AqpZ. However, subsequent studies have shown that the gating phenomenon is caused by perturbation of a non-native detergent environment [92]. Solid-state NMR examination found that in the synthetic bilayers and the native cytoplasmic membrane of *E. coli*, a permanent open conformation was found for the R189 side chain of AqpZ, ruling out the gating mechanism in AqpZ regulation.

Currently, researchers are exploring other possible regulatory mechanisms that modulate the activity of prokaryotic aquaporins. A recent study found that Trp219, a residue extremely conserved within loop E of aquaglyceroporins, impacts the function and oligomerization of the *E. coli* GlpF [93]. Trp219 deeply protrudes into the GlpF protein and interacts with the adjacent loops; therefore, this residue is key to a defined vestibule structure and glycerol accumulation, and the stability of the active GlpF tetramer. Substitution of Trp219 decreases the activity of GlpF and impairs the stability of the tetrameric protein. In addition, a recent study showed that anionic lipids modulate the activity of the aquaglyceroporin GlpF by stabilizing their tetrameric structure [94], which is important for glycerol transport, as increasing fractions of the oligomerization-impaired mutant GlpF E43A affect the activity of the GlpF heterotetramer [95]. AqpZ is also stabilized by various types of lipids, with cardiolipin imparting the most significant resistance to unfolding [96,97]. These observations illustrate a potential mechanism by which the activity of an α-helical membrane protein is modulated by the negative charge density around the protein.

## 8. Future Research Directions

Although investigations on prokaryotic aquaporins are far behind those of eukaryotic species, the current information in a handful of species has shown that prokaryotic aquaporins have a wide range of physiological functions. For convenience of comparison, Table 1 summarized the transport characteristics and physiological functions of prokaryotic and some eukaryotic aquaporins. Moreover, sequence alignment of representative prokaryotic aquaporins shows differential SF amino acid residues (Figure 4). Given that the amino acid characterization and pore diameter of the SF region determine substrate specificity, variations in the substrates of prokaryotic aquaporins are implied. Furthermore, the orthodox water and glycerol-type aquaporins, such as the *S. oligofermentans* So-AqpA and the *L. plantarum* GlpF, have been verified to facilitate other small molecules or solutes across the cellular membrane. Considering the unparalleled diversity of prokaryotic species, intensive interrogation into the prokaryotic aquaporins would expand the current substrate categories.

The majority of the prokaryotic microbes are unicellular organisms with no cell departmentalization; thus, extracellular substances entering cells and the efflux of the endogenous metabolites could be one of the important approaches for cell survival and fitness. Thus, the functions of aquaporins could be more important for prokaryotes. An exemplified bacterial AQP is the streptococcal peroxiporin for the efflux of the by-product H_2_O_2_, which not only plays a detoxification role but also endows the bacterium with intra or inter-species competitiveness. By understanding AQP-related bacterial survivability and adaptability either in natural environments or within infected hosts, one could control bacteria via drug design by targeting their AQPs.

Microorganisms inhabit diverse environments, and thus, have developed multiple approaches to cope with fluctuations in environmental factors. Prokaryotic AQPs have been demonstrated to help bacteria deal with the osmotic and oxidative stress and nutritional fluctuations. Moreover, expressions of these aquaporin genes are regulated at transcriptional levels in response to external stimuli. However, whether prokaryotic organisms also employ gating or trafficking mechanisms to regulate aquaporins’ expressions, requires further investigation. Answering these questions would improve our understanding of the regulatory mechanisms of MIP family proteins.

## Figures and Tables

**Table 1:** The transport characteristics and physiological functions of aquaporin homologues in representative eukaryotic and prokaryotic organisms.

Organism	Gene Number	Uniprot Accession Number	Transported Substrate	Heterologously Expressed Host	Expression Sites in Itself	Suggested Physiological Functions	References
Eukaryote							
Human							
AQP0	4284	P30301	Water	Not determined	Lens	congenital cataract (loss of function mutation)	[32,98]
AQP1	358	P29972	Water, O_2_, H_2_O_2_, CO_2_, NO	*Xenopus* oocytes; yeast;	Renal tubules, red blood cells	Water permeability	[1,25,32,98]
AQP2	359	P41181	Water	Not determined	Renal collecting duct	nephrogenic diabetes insipidus (loss of function mutation)	[32,98]
AQP3	360	Q92482	Glycerol, urea, H_2_O_2_, water	Not determined	Renal collecting duct, adipocytes	Glycerol permeability	[11,27,32,98]
AQP4	361	P55087	Water, CO_2_, NO, O_2_	Not determined	Astrocytes	Cerebrospinal fluid flux	[32,98]
AQP5	362	P55064	Water, H_2_O_2_, CO_2_	Not dermined	Glandular tissues such as salivary gland	Saliva secretion	[32,98]
AQP6	363	Q13520	Water, nitrate	Not determined	Intracellular vesicles in renal collecting duct	Acid secretion	[32,98]
AQP7	364	O14520	Glycerol, urea, water	Not determined	Fat cells, renal proximal tubule	Glycerol permeability	[11,32,98]
AQP8	343	O94778	Water, H_2_O_2_	Yeast	Intestinal epithelium	None identified	[27,32,76,98]
AQP9	366	O43315	Glycerol, urea, H_2_O_2_, water	Not determined	Hepatocytes, erythrocytes	Glycerol permeability	[11,32,98]
AQP10	89872	Q96PS8	Glycerol, urea, water	Protepolymersome, Yeast	adipose tissue	None identified	[11,98,99]
AQP11	282679	Q8NBQ7	Water	Proteoliposome	Liver, testis	None identified	[32,100]
AQP12	375318	Q8IXF9	Unknown	Not dermined	Exocrine pancreas	None identified	[32,100]
Plant							
*Nicotiana tabacum*	NtAQP1	Q9ZR68	CO_2_	*Xenopus* oocytes	Leaf	Increasing leaf growth	[23]
	PIP1;3	Q40595	O_2_	Yeast	Root	Increase of ATP levels in the apical root segments	[25]
*Arabidopsis thaliana*	NIP1;2	Q8LFP7	Aluminum-malate	Yeast	Root	Aluminum uptake, translocation and tolerance	[24]
	PIP2;1	P43286	H_2_O_2_	Not determined	Guard cell	Stomatal closure	[13]
Prokaryote							
Bacteria							
*Escherichia coli*	*aqpZ*	P60844	Water	*Xenopus* oocytes	Cellular membrane	Osmostic stress resistance	[39,44,80]
	*glpF*	P0AER0	Glycerol	*Xenopus* oocytes	Cellular membrane	Growth on low concentration glycerol	[40,70]
*Streptococcus oligofermentans*	*aqpA*	I872_01445	H_2_O_2_	*E. coli*, Yeast	Cellular membrane	H_2_O_2_ detoxification and interspecies competition	[35]
*Lactobacillus plantarum*	*glpF1*	F9UST3	Lactic acid, urea, H_2_O_2_	*Xenopus* oocytes, Yeast	Cellular membrane	Lactic acid stress tolerance	[84]
	*glpF2*	F9USY3	Water, glycerol, dihydroxyacetone, H_2_O_2_	*Xenopus* oocytes, Yeast	Cellular membrane	None identified	[84]
	*glpF3*	F9UTW9	Water, glycerol, dihydroxyacetone, H_2_O_2_	*Xenopus* oocytes, Yeast	Cellular membrane	None identified	[84]
	*glpF4*	F9UMX3	Water, glycerol, dihydroxyacetone, lactic acid, urea, H_2_O_2_	*Xenopus* oocytes, Yeast	Cellular membrane	Lactic acid stress tolerance	[84]
*Pseudomonas aeruginosa*	*glpF*	Q51389	Glycerol	Not determined	None identified	Growth on glycerol	[83]
Archaea							
*Methanothermobacter marburgensis*	*aqpM*	Q9C4Z5	Water, glycerol	Proteoliposomes	Cellular membrane	None identified	[45,51,52]
*Archaeoglobus fulgidus*	AfAQP	O28846	Water	Proteoliposomes	Cellular membrane	None identified	[50]
